# Tumor-antigen-specific polyclonal antibodies suppress tumor growth and metastasis in heterogeneous solid tumors

**DOI:** 10.3389/fonc.2026.1947593

**Published:** 2026-10-09

**Authors:** Carine Ciron, Ophélie Dauphouy, Pierre Morice, Pierre-Joseph Royer, Gwenaëlle Evanno, Juliette Rousse, Françoise Shneiker, Bernard Vanhove, Odile Duvaux, Firas Bassissi

**Affiliations:** 1 Xenothera, Nantes, France; 2 Nantes Université, Institut National de la Santé et de la Recherche Médicale (INSERM), Center for Research in Transplantation and Translational Immunology (CR2TI), Nantes, France

**Keywords:** annexin A2, CKAP4, immunotherapy, metastasis, polyclonal antibodies, SLC3A2, tumor microenvironment

## Abstract

**Introduction:**

Tumor heterogeneity and signaling redundancy limit the efficacy of monospecific antibody therapies in solid malignancies. Here we used our platform of glyco-humanized polyclonal antibodies (GH-pAb), to investigate whether targeting multiple tumor-associated antigens provides integrated anti-tumor activity.

**Methods:**

GH-pAb (hereinafter XON7) were generated by immunization of double-knockout pigs with human tumor cells. Antigen specificity of XON7 was assessed using a human proteome array and validated by ELISA. Anti-tumor mechanisms were evaluated *in vitro* on 2D or 3D-cultures. Efficacy was tested *in vivo* in cell or patient-derived xenograft (CDX, PDX), in chicken chorioallantoic membrane (CAM) model, and in orthotopic/metastatic triple negative breast cancer (TNBC) models.

**Results:**

XON7 demonstrated tumor-restricted binding across tissue microarrays without detectable staining in matched normal tissues. Mechanistically, XON7 induced potent cytotoxicity across a wide tumor panel through complement activation, apoptosis, and antibody-dependent cellular phagocytosis. Proteome profiling identified predominant reactivities against solute carrier family 3 member 2 (SLC3A2), annexin A2 (ANXA2), and cytoskeleton-associated protein 4 (CKAP4), all broadly expressed tumor-associated surface proteins. Immunodepletion experiments revealed that concurrent targeting of these three targets was required to maintain the full anti-proliferative activity of XON7, supporting a multitarget-dependent mechanism. XON7 also induced released of high-mobility group protein box 1 (HMGB1), a hallmark of immunogenic cell death. *In vivo*, XON7 inhibited tumor growth in CDX and PDX models, and markedly reduced metastatic dissemination in orthotopic and intravenous TNBC models. In a CAM model, XON7 synergized with PD-1 blockade, enhancing tumor control and promoting macrophage polarization toward a M1 phenotype.

**Conclusion:**

By simultaneously engaging non-redundant tumor pathways and reshaping the tumor microenvironment, XON7 may overcome key resistance mechanisms of solid tumors.

## Introduction

The vertebrate immune response is inherently polyclonal, generating high-affinity antibodies directed against multiple epitopes of a given antigen and activating diverse effector mechanisms including complement-dependent cytotoxicity (CDC), antibody-dependent cellular cytotoxicity (ADCC), antibody-dependent cellular phagocytosis (ADCP) or apoptosis (1). Historically, polyclonal antibodies (pAbs) raised in animals were explored as experimental anti-cancer therapies, including early studies in osteosarcoma that reported substantial tumor regression (2). More recently, therapeutic pAbs have demonstrated clinical utility in transplantation immunosuppression, infectious diseases, anti-toxin therapy, and envenomation (3). However, concerns related to their immunogenicity and toxicity following repeated administration limited their development in oncology, where monoclonal antibodies (mAbs) have become the dominant antibody-based therapeutic modality.

Over the past three decades, more than sixty monoclonal antibodies have been approved for cancer treatment, producing significant improvements in patient outcomes across hematologic malignancies and solid tumors (4). Despite these advances, monospecific targeting faced limitations which became increasingly evident. Antigen loss, tumor escape, clonal selection, and signaling redundancy frequently undermine therapeutic durability (5, 6). In addition, spatial and temporal tumor heterogeneity promotes the emergence of resistant subclones capable of bypassing single-epitope targeting strategies (7, 8). To address this challenge, engineered bispecific and multispecific antibodies have been developed to simultaneously engage multiple targets. Although conceptually attractive, these formats remain technically complex, costly to manufacture, and constrained by structural and pharmacokinetic limitations (9, 10).

Polyclonal antibody therapeutics may provide an alternative strategy to address tumor heterogeneity. By simultaneously recognizing multiple epitopes and potentially multiple antigens, polyclonal preparations may limit escape mechanisms while engaging diverse immune effector pathways. Recent advances in transgenic animal platforms and glycoengineering, including the development of swine lacking immunogenic xenoantigens such as α1,3 galactose (α-Gal) and N-glycolylneuraminic acid (Neu5Gc), have substantially reduced the historical limitations associated with pAbs and enabled the production of reproducible glyco-humanized pAb (GH-pAb) (11–13).

XON7 is a swine GH-pAb generated against human tumor cells. We investigated here whether targeting multiple tumor-associated antigens by XON7 could provide integrated anti-tumor activity. We showed that XON7 exerted significant *in vitro* and *in vivo* anti-tumor effects on tumor growth and metastatic dissemination and synergized with immune checkpoint inhibition. The simultaneous targeting of several tumor proteins can regulate complementary aspects of tumor biology, including metabolic alteration, survival signaling, and tumor–microenvironment interactions. Preservation of multi-targeting contributes to the anti-tumor activity of XON7 and supports polyclonality as a strategy to address tumor heterogeneity in solid malignancies.

## Materials and methods

### Animals and cell lines

Seven-week-old female NMRI nude mice were obtained from Janvier Labs (Le Genest-Saint-Isle, France). Seven-week-old female SRG rats were obtained from Charles River (Saint-Germain-Nuelles, France). Double-KO pigs for cytidine monophosphate-N-acetylneuraminic acid hydroxylase and α1,3-galactosyl-transferase genes were generated and bred as previously described (14). All procedures involving the use of animals in this study were carried out in accordance with EU directive 2010/63/EU, which regulates animal experimentation and have been approved by “Comité d’Ethique Pays de La Loire” (CEEA PdL n.6) and by the “Direction Générale de la Recherche et de l’Innovation” of the French Ministry “Enseignement Supérieur, de la Recherche et de l’Innovation”: APAFIS 29523, APAFIS 38649. All methods are reported in accordance with ARRIVE guidelines. All animal experiments were conducted in strict adherence to the principles of the 3Rs (Replacement, Reduction, and Refinement), ensuring ethical and human treatment throughout the study. Tumoral cell lines LNCaP (ACC 256), HCT-116 (ACC 581), KMS-12-BM (ACC 551), A549 (ACC 107), H460 (ACC 737), Hep-G2 (ACC 180) and SK-MEL-30 (ACC 151) were purchased from Deutsche Sammlung von Mikroorganismen und Zellkulturen (DSMZ, Braunschweig, Germany) and MBA-MB-231 (HTB-26) were purchased from American Type Culture Collection (ATCC, Manassas, VA, USA). Peripheral blood mononuclear cells were obtained from healthy donors at Etablissement Français du Sang (EFS, Nantes, France). The PDX cell lines used in this study were owned by Charles River Laboratory (Wilmington MS, USA) or Champion Oncology (Rockville, MD, USA). Informed consent was obtained from all subjects and/or their legal guardians prior to tissue collection, as per the requirements of the institutional and regulatory framework.

### Antibody generation and target profiling

XON7 was generated by immunizing double-KO pigs with a human tumor cell line presenting aberrant expression of multiple cancer-associated antigens. Immunizations followed a procedure developed by Xenothera (Nantes, France). Purification of the IgG fraction by protein A chromatography followed GMP and ICH guidelines. Antigen specificity was determined using a human proteome microarray (HuProt™, CDI LABS, Baltimore, MD) as per manufacturer instructions.

### Depletion of SLC3A2, CKAP4 and Annexin A2 specific IgG fractions

SLC3A2-specific or SLC3A2 depleted fractions were isolated from the XON7 polyclonal antibody by Rapid Novor (Kitchener, Canada). CKAP4- and Annexin A2 depleted fractions were isolated from the XON7 polyclonal antibody using biotinylated recombinant human CKAP4 (Kactus Bio, CAP-HE1P4B) or biotinylated recombinant human Annexin A2 (RayBiotech) immobilized on streptavidin magnetic beads (Dynabeads™ MyOne™ Streptavidin C1, Thermo Fisher). Briefly, 20 µL of Dynabeads were washed three times with PBS, then incubated with 30 µg of biotinylated antigen in 200 µL PBS for 1 hour at room temperature with rotation. Beads were washed three times in PBS containing 0.01% Tween-20 with a final wash in PBS to remove unbound protein, then incubated with 4 mg of XON7 in 500 µL PBS for 1 hour at +4 °C with rotation. After binding, beads were washed three times with PBS to collect non-specific IgG. Insufficient quantities of CKAP4 and Annexin A2 specific fractions could be obtained. ELISA were performed to verify the depletion of each fraction and the enrichment of SLC3A2 specific fraction (**Supplementary Figure 1**).

### HMGB1 quantification

Tumor cells (MDA-MB-231, HCT-116 or A549) were plated in 96-well plates for 24h to let them attach and treated with XON7, SLC3A2 specific fraction, or SLC3A2-depleted fraction at the indicated concentrations. After 24h, HMGB1 release was measured using the Lumit^®^ HMGB1 Human Immunoassay (Promega) according to the manufacturer’s instructions. Luminescence was recorded using a GloMax^®^ Discover Microplate Reader (Promega).

### 2D functional assays

Complement-dependent cytotoxicity (CDC), apoptosis, antibody-dependent cell-mediated cytotoxicity (ADCC), and antibody-dependent cell-mediated phagocytosis (ADCP) were performed using standard protocols. Briefly, tumor cells or PBMCs were treated with varying concentrations of XON7. CDC was measured by AO/DAPI staining after 6 hours incubation in the presence of 15% rabbit complement (CedarLane Labs). This complement concentration was chosen as it does not induce cells lysis in the absence of XON7. ADCC used CFSE-labeled tumor cells and human NK cells (E:T = 4:1), ADCP used CFSE-labeled tumor cells and CD68+ macrophages differentiated from monocytes (E:T = 4:1). Readouts were performed on a Nucleocounter NC-3000 image cytometer (ChemoMetec, Lillerød, Danemark) after 6h of incubation. For apoptosis, tumor cells cultured with XON7 for 24h were collected, washed twice with DMEM 10% FCS and labeled with CF488-conjugated Annexin V (Biotium, Freemont, CA, USA) for 15 min at 37 °C. After two washes, cells were stained with 10 µg/mL propidium iodide (PI) (Chemometec) before analysis on a Nucleocounter^®^ NC3000 cytometer. Percentages of cells in early apoptosis (Annexin V+/PI− cells) and in late apoptosis (Annexin V+/PI+) were added to determine the overall % of apoptosis. Cell viability of PDX-derived cell lines was performed by Charles River Laboratories. Briefly, cells were exposed to XON7 (0.3–1000 µg/mL) for 24 h in presence of rabbit complement, and viability was assessed with the CellTiter-Glo^®^ assay (Promega). Luminescence was measured using the EnVision^®^ Xcite plate reader (PerkinElmer).

### 3D spheroid assays

Tumor spheroid assays were performed using A549, HCT-116 and MDA-MB-231 cancer cell lines seeded at 1000 cells/well in Nunclon Sphera 96-well round-bottom plates. Plates were then centrifuged to promote aggregation and incubated for 3 days to allow spheroid formation. For resistance testing, HCT-116 spheroids were pretreated with XON7 (100 or 300 µg/mL) for 24h. On day 3, a second dose of XON7 was added to some groups. Spheroid viability was assessed on day 10 using the CellTiter-Glo^®^ 3D assay (Promega), and luminescence measured with a GloMax reader (Promega).

### Mouse xenograft models

Subcutaneous tumors were established by injecting 5×10^6^ human tumor cells (A549, LNCaP, HCT-116, or KMS-12-BM) into the flank of NMRI nude mice (Janvier) (n = 8/group). When tumors reached 50 mm³, XON7 was administered intraperitoneally (35 mg/kg, twice a week for 4 weeks). Tumor growth was monitored using a caliper. Tumor volume was calculated using the standard formula: 1/2 ×Width ×Width × Length, assuming a standard and constant tumor shape.

### Patient derived xenograft models

Two dedifferentiated liposarcoma PDX models (CTG-2309 and CTG-0910) were performed by Champions Oncology (Hackensack, NJ). PDX were implanted subcutaneously in Athymic Nude NU(Ncr)-Foxn1^nu mice. Treatments began when tumor volumes reached 150–200 mm³. Groups received biweekly intraperitoneal injection of PBS (10 mL/kg) or XON7 (40 mg/kg). Tumor volumes were monitored by calipers and calculated using the standard formula 1/2 ×Width ×Width × Length, assuming a standard and constant tumor shape.

### In ovo chick chorioallantoic membrane xenograft model

Chick chorioallantoic membrane (CAM) xenograft model was performed by Inovotion (La Tronche, France). Fertilized White Leghorn eggs were incubated at 37.5 °C until embryonic day 9 (E9) where H460 human lung cancer cells (1×10^6^) were grafted onto the CAM. On E10, treatments were initiated as per **Table 1**. On E18, tumors were harvested and weighed. Metastatic invasion was assessed by qPCR of human Alu sequences in distal CAM and brain tissues. Immune cell polarization was analyzed from tumor RNA by RT-qPCR (chicken iNOS2, CD86, CD80, CCL17), normalized to GAPDH.

**Table 1:** Groups and treatments for chorioallantoic membrane xenograft model CAM model.

Group	Cpd/vehicle	Inj. vol (µl)	Injected	In ovo final	Dosing regimen
CTRL	50% rabbit serum	200	–	–	E10, E12, E14, E15, E17
Anti-PD1	Pembrolizumab	200	0.75mg/ml	2.5mg/ml	E10, E12, E14, E15, E17
XON7	XON7	200	3mg/ml	10mg/ml	E10, E12, E14, E15, E17
XON7 + Anti-PD1	XON7 + Pembrolizumab	200	3mg/ml + 0.75mg/ml	10mg/ml + 2.5mg/ml	E10, E12, E14, E15, E17

### Orthotopic and metastatic triple negative breast cancer (TNBC) models

Luciferase-expressing MDA-MB-231, human triple negative breast cancer cells were injected either orthotopically into the mammary fat pad (orthotopic model) or intravenously (metastatic model) in immunodeficient SRG rats. For orthotopic TNBC model, XON7 (40 mg/kg, intraperitoneal injection, twice a week) was initiated upon tumor palpation (around 50mm^3^). Tumor volumes were monitored bi-weekly. For metastatic TNBC model, treatment started at day 1 post-injection. Metastatic burden was monitored by bioluminescence imaging three times per week.

### Immunohistological analyses and tissue microarray (TMA)

Tissue microarrays (Oncotest™, Charles River) were stained with XON7 (5 µg/mL), followed by goat anti-pig HRP-conjugated secondary antibody (Mabtech AB) and ImmPACT^®^ VIP (Vector Labs). Signal quantification was performed using ImageJ. Number of stained biopsies and percentage of positive areas in positive biopsies were determined.

### Statistical data analysis

Experiments were performed at least three independent times unless otherwise indicated, with the number of replicates specified in the corresponding figure legends. Tumor growth and metastatic burden were analyzed using non-parametric tests and are presented as medians with 95% confidence intervals. Comparisons between groups were performed using one-way ANOVA followed by Dunn’s multiple comparisons test or Dunnett’s test when comparing treatment groups with a control condition. Tumor growth curves were analyzed using two-way repeated-measures ANOVA. Combination effects were evaluated using the Bliss independence model (expected effect = E_a + E_b − E_a × E_b). Statistical analyses were performed using GraphPad Prism version 8 (GraphPad Software) and XLSTAT. Differences were considered statistically significant when p < 0.05.

## Results

### Tumor type recognition and selectivity

Tumor selectivity was first evaluated by immunohistochemistry on a tissue microarray comprising ten tumor types paired with matched healthy tissues. XON7 showed staining across multiple malignancies, including breast, pancreatic, prostate, melanoma, non-Hodgkin lymphoma, and gastric cancers, whereas no detectable staining was observed in corresponding normal tissues (**Table 2**). Analysis of an extended cohort of tumor biopsies confirmed robust binding across several cancer types (**Table 3**). High staining intensity was observed in small-cell lung cancer (84.5% ± 6.6%), pleural mesothelioma (85.2% ± 5.1%), and leukemia (84.9%). Non-small-cell lung cancers also exhibited strong but more heterogeneous staining (respectively 69.7% ± 3.4% and 70.8% ± 7.1% for epidermoid and large cell subtype). Moderate yet steady binding was detected in pancreatic (24.2% ± 3.1%) and triple-negative breast cancers (14.5% ± 4.0%). In contrast, osteosarcoma, gastrointestinal stromal tumors, and thyroid cancers showed greater inter-sample variability, consistent with heterogeneity in antigen expression. Together, these results indicate that XON7 exhibits reproducible multi-tumor binding with selective reactivity toward malignant tissues.

**Table 2:** Tumoral specificity of XON7 assessed by tissue microarray.

Tissues	Diagnostic	XON7 binding
Brain	healthy	-
	glioblastoma	-
Breast	healthy	-
	Invasive ductal carcinoma	+
Muscle	healthy	-
	leiomyosarcoma	-
Pancreas	healthy	-
	adenocarcinoma	+
Prostate	healthy	-
	adenocarcinoma	+
Skin	healthy	-
	melanoma	+
Intestine	healthy	-
	Non-Hodgkinien lymphoma	+
Stomach	healthy	-
	adenocarcinoma	+
Ovary	healthy	-
	adenocarcinoma	-
Uterus	healthy	-
	adenocarcinoma	-

**Table 3:** XON7 cross-reactivity on human cancer biopsies.

Human cancer biopsies	Number of XON7 stained patient’s biopsies/number of tested biopsies	Mean % of XON7 stained area in positive biopsies
Asian colon cancer	4/4	86.83% +/- 4.12%
Colon cancer	45/63	19.52% +/- 2.57%
Prostate cancer	1/1	19.23%
Breast cancer HER2	4/7	6.75% +/- 3.6%
Breast cancer triple negative	10/12	14.49% +/- 4%
Ovarian cancer	13/16	10.42% +/- 2.6%
Testicular cancer	1/2	18.33%
Uterus cancer	1/1	24.3%
Thyroid cancer	3/4	43.38% +/- 24.15%
Xenograph of asian ethnicity	1/1	75.3%
Non-small cell lung cancer epidermoid	29/30	69.7% +/- 3.4%
Non-small cell lung cancer large cell	9/10	70.8% +/- 7.1%
Small cell lung cancer	9/9	84.5% +/- 6.6%
Pleura-mesothelioma	9/9	85.2% +/- 5.1%
Cancer of the central nervous system	13/13	37.01% +/- 5.5%
Gastrointestinal stromal cancer	2/2	64.65% +/- 25.1%
Epidermal carcinoma	1/1	48.15%
Sarcoma of Asian ethnicity	1/1	58.3%
Osteosarcoma	3/4	58.41% +/- 17.7%
Soft tissue sarcoma	10/10	40.15% +/- 9.2%
Leukemia	1/1	84.85%
Acute lymphoblastic leukemia	1/1	83.14%
Acute myeloid leukemia	10/10	79.36% +/- 5.4%
Diffuse large B cell lymphoma	7/7	70.5% +/- 9.7%
Pancreatic cancer	35/48	24.2% +/- 3.1%

### XON7 displays broad cytotoxic activity across diverse tumor models

The cytotoxic activity of XON7 was evaluated across a panel of 85 tumor cell lines or PDX representing fifteen cancer types, including both established and patient-derived models (**Figure 1**). XON7 induced effective dose-dependent cytotoxicity in most of the tested models. In triple-negative breast cancer, sarcoma, colon cancer, gastric cancer and prostate cancer models, moderate concentrations (≤100µg/ml) frequently induced 50% cytotoxicity, and higher doses (≥300µg/ml) more than 80% cytotoxicity. Noteworthy, the concentrations tested are compatible with the plasma concentrations reached in patients treated with XON7 (clinical trial NCT06154291 and (14)). Intermediate activity was observed in melanoma, liver cancer, ovarian cancer, bladder cancer, leukemia, multiple myeloma, and pleural mesothelioma models. Cell lines derived from non-small-cell lung cancer, pancreatic cancer, and head-and-neck cancers displayed more diverse responses, consistent with variability in antigen expression and tumor heterogeneity.

**Figure 1:**
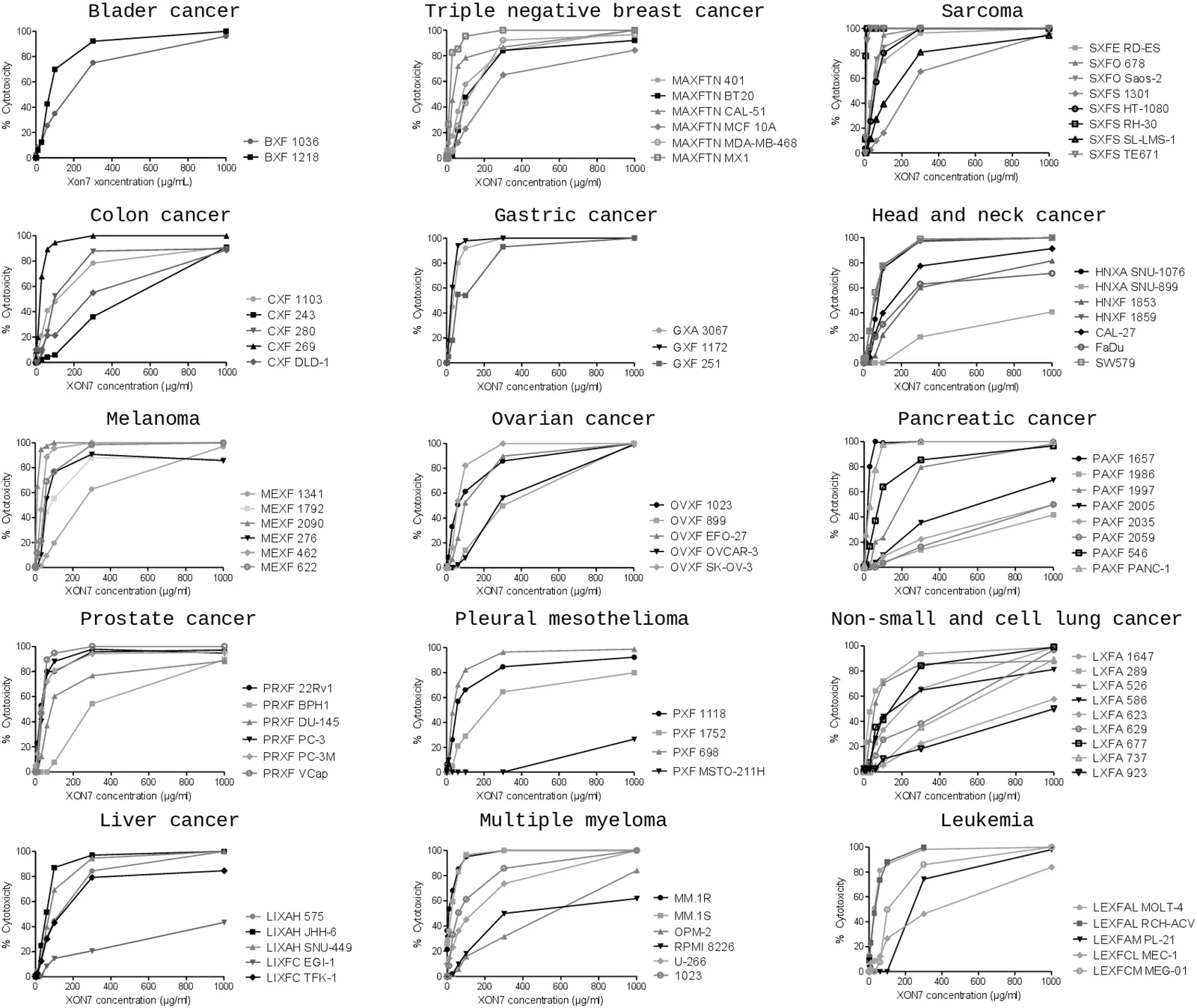
*In vitro* cytotoxicity of XON7 across diverse cancer types. Tumor cell lines derived from solid and liquid cancers were treated with increasing concentrations of the therapeutic polyclonal antibody XON7 (0–1000 µg/mL), and cytotoxicity was measured as a percentage relative to untreated controls after 24h of incubation (Cell-Titer Glo assay). Each panel represents a specific cancer type, and corresponding cell lines, as indicated.

### XON7 exhibits consistent and reproducible multi-target interaction

The dominant antigen specificity of XON7 was assessed by proteome-scale screening using a human protein array comprising more than 21,000 recombinant proteins. Among the 10 top Z-scored targets, we focused on three corresponding to prominent tumor-associated surface proteins: SLC3A2 (also known as CD98hc), CKAP4 and Annexin A2 (**Table 4**). Clinical relevance of these targets was investigated in 39 human cancer types using the TIMER and GEPIA platforms (15, 16). *SLC3A2*, *CKAP4* and *ANXA2* were upregulated in respectively 16, 14 and 16 cancer types compared to normal tissues (**Supplementary Figure 2**). A negative impact on the clinical outcome was suggested in cancer such as bladder carcinoma, head and neck carcinoma, glioma, liver hepatocarcinoma, lung adenocarcinoma or mesothelioma (**Supplementary Figure 3**). To assess XON7 reproducibility, 3 independent XON7 batches were prepared and tested for their ability to bind to *SLC3A2*, *CKAP4* and *ANXA2*. All batches displayed comparable binding profiles against these targets (**Supplementary Figure 4**) and similar cytotoxic activities across multiple tumor cell lines (**Supplementary Figure 5**), showing a strong inter-batches comparability of XON7 preparation.

**Table 4:** Predominant 3 targets of XON7 identified by protein array.

XON7 target	Z score	Expression	Function	Reference
SLC3A2	47.3	High, overexpressed in many solid tumors	Amino acid transporter subunit + integrin co-receptor; promotes proliferation, survival, metastasis	SLC7A11: the Achilles heel of tumor? Jiang Y, Sun M. Front Immunol. 2024 Jul 8;15:1438807. doi: 10.3389/fimmu.2024.1438807.
CKAP4	31.6	Moderate to high (if DKK1+)	Receptor for DKK1; activates PI3K/AKT signaling; promotes proliferation	The Emerging Role of CKAP4 in GI Cancer: From Molecular Pathways to Clinical Applications. Despotidis M, Lyros O, Driva TS, Sakarellos P, Thieme R, Mamilos A, Sakellariou S, Schizas D. Curr Oncol. 2025 Oct 7;32(10):561. doi:10.3390/curroncol32100561.
ANXA2	26.8	High in aggressive cancers	Plasminogen receptor; promotes invasion, angiogenesis, metastasis	Annexin A2: the feasibility of being a therapeutic target associated with cancer metastasis and drug resistance in cancer microenvironment. Weijie S. Discov Oncol. 2024 Dec 18;15(1):783. doi: 10.1007/s12672-024-01693-8.

### XON7 engages multiple effector pathways and requires multiple antigen engagement for anti-tumor activity

Functional assays revealed that XON7 triggers strong complement-dependent cytotoxicity (CDC) and apoptosis in tumor cells, while showing minimal activity toward healthy peripheral blood mononuclear cells (PBMCs) (**Figures 2A, B**). A very weak cell death (less than 10%) was obtained by ADCC (**Figure 2C**), as expected for a swine GH-pAb (17), whereas antibody-dependent cellular phagocytosis (ADCP) was observed (**Figure 2D**). Altogether, these findings indicate that CDC, apoptosis and to a lesser extend phagocytosis represent the predominant effector mechanisms associated with XON7 activity. To determine whether XON7 activity depended on multiple antigen targeting, we compared native XON7 with fractions depleted of SLC3A2, Annexin A2 or CKAP4 antibodies, or combination thereof. Native XON7 significantly inhibited A549, HCT-116, and MDA-MB-231 proliferation compared to control, while single-antibody depleted preparations displayed a limited reduction in cell growth (**Figure 3**). These observations indicate that polyclonality contributes substantially to the anti-proliferative activity of XON7.

**Figure 2:**
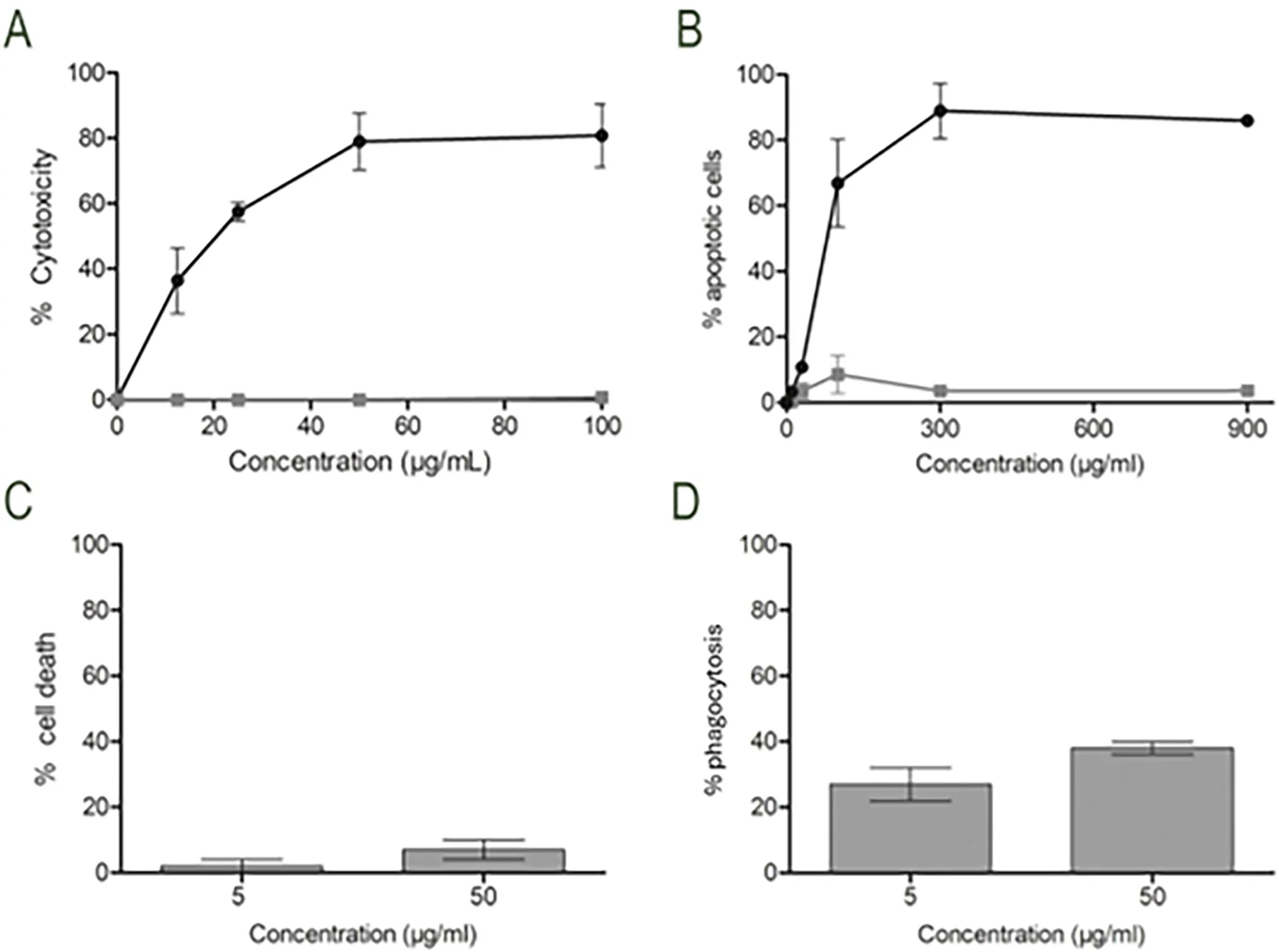
Specific cytotoxic activity of XON7 against tumor cell line via several mechanisms of action. **(A)** Dose-dependent CDC activity in the KMS-12-BM tumor cell line (dark line) without cross-reactivity in PBMCs of healthy donors (gray line). **(B)** Dose-dependent apoptosis in the KMS-12-BM tumor cell line (dark line) without cross-reactivity in PBMC of healthy donors (gray line). Investigation of ADCC **(C)** and phagocytosis **(D)** on KMS-12-BM. All data are expressed as means ± SEM; n=3.

**Figure 3:**
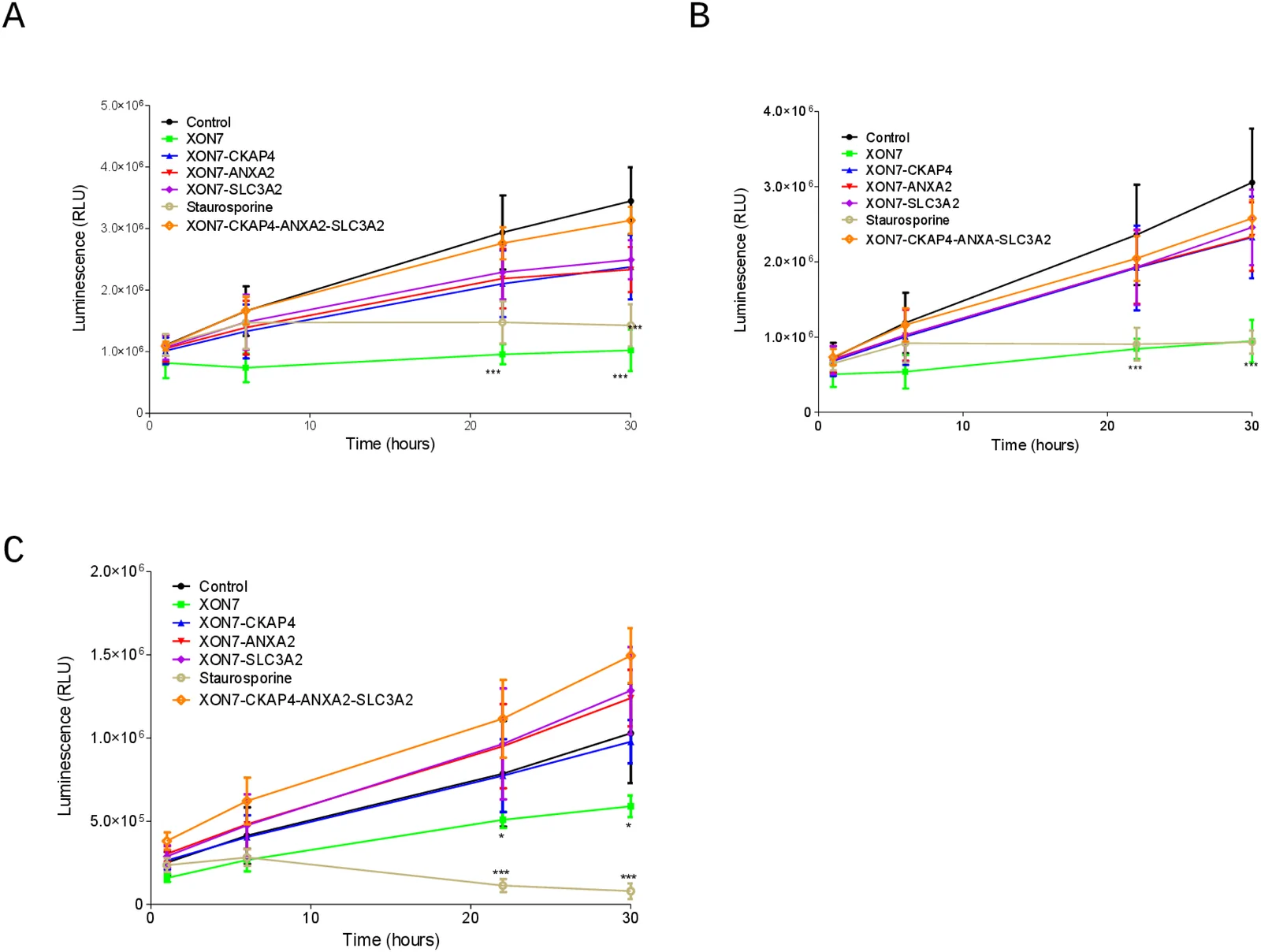
The anti-proliferative activity of XON7 depends on simultaneous engagement of its three tumor-associated targets. Comparative cell-proliferation assays in A549 [NSCLC, **(A)**], HCT-116 [colon carcinoma, **(B)**], and MDA-MB-231 [triple-negative breast cancer, **(C)**] cells treated with XON7 or a depleted preparation lacking antibodies to SLC3A2, Annexin A2, and CKAP4. Luminescence-based viability was monitored over time and expressed as relative luminescence units (RLU). Staurosporin was used as a positive control. Data represent mean ± SEM, n=3. One-way Anova, *post hoc* test Fischer, *p<0.05; *** P < 0.001.

### SLC3A2 engagement contributes to tumor stress induction

Targeting SLC3A2 may lead to the release of damage associated molecular patterns (DAMP) such as HMGB1 (18). We thus investigated whether XON7 induced cellular stress pathways associated with tumor damage signaling. Treatment with native XON7 triggered extracellular release of HMGB1 by HCT-116, MDA-MB-321 and A549 tumor cells (**Figure 4**). These effects were reproduced by the SLC3A2-specific antibody fraction and were abolished when SLC3A2-reactive antibodies were depleted.

**Figure 4:**
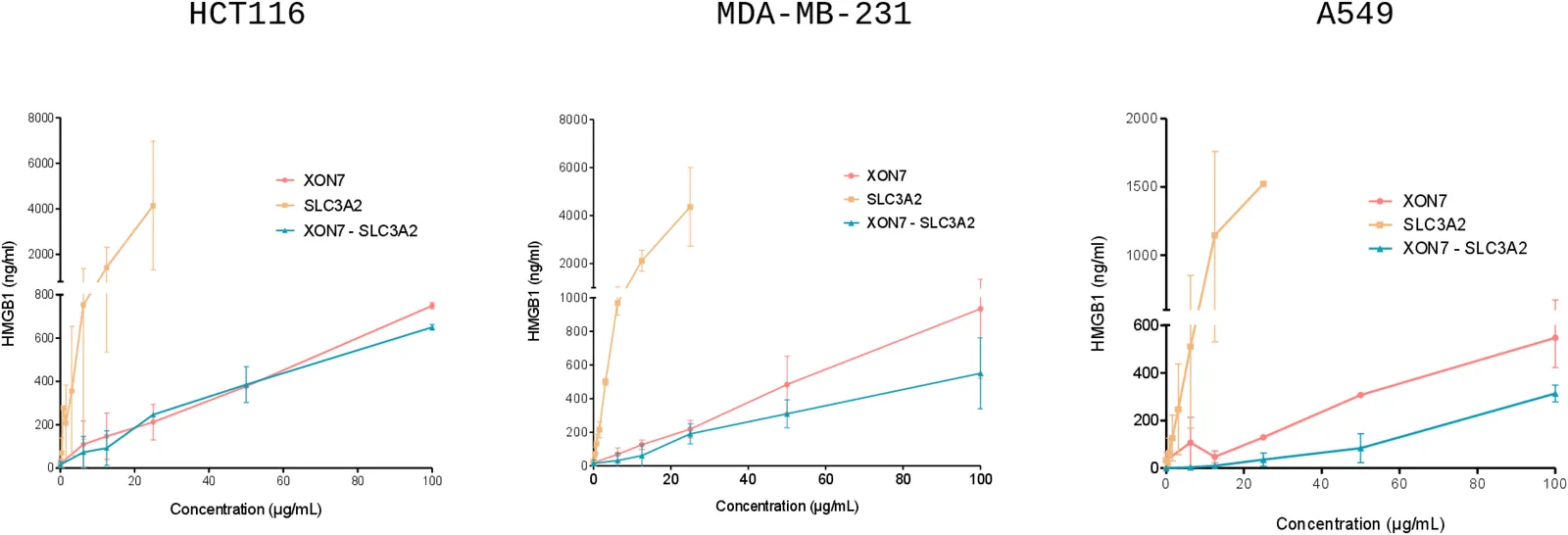
SLC3A2 engagement by XON7 induces release of HMGB1. HCT-116, MDA-MB-231 and A549 cells were treated with native XON7, the SLC3A2-specific fraction isolated from XON7, or the SLC3A2-depleted preparation. Quantification of HMGB1 release after 24h of treatment. Data are shown as mean ± SEM, N = 2.

### XON7 maintains its activity after repeated treatments in 3D tumor models and suppresses tumor cell invasion

To evaluate sustainability of anti-tumor activity and potential resistance emergence, repeated treatment experiments were conducted in various cancer cell lines (**Figure 5**). In KMS-12-BM myeloma cells in suspension, repeated XON7 administrations induced replicated cytotoxicity without evidence of resistance (**Figure 5A**). Neither reduction of tumor cell lysis, nor acceleration of tumor cell regrowth could be observed after four XON7 treatments. XON7 activity was also maintained in 3D-spheroid models. Reduction of HCT-116 colon cancer spheroid growth by 43.5% and 69.6% was observed at days 4 and 7 (**Figure 5B**). In A549 NSCLC cells, 70% spheroid volume reduction was maintained until day 11 after a single XON7 treatment (**Figure 5C**). In MDA-MB-231 triple-negative breast cancer spheroids, XON7 inhibited spheroid expansion and reduced cell viability, with repeated treatments producing stronger effects than paclitaxel (**Figure 5D**). Pretreatment experiments revealed cell-type–specific responses. In MDA-MB-231 cells, XON7 pretreatment impaired spheroid-forming capacity, suggesting inhibition of tumor-initiating potential (**Figures 5E, F**). In contrast, HCT-116 cells retained spheroid-forming ability but became more sensitive to subsequent XON7 exposure (**Figure 5G**).

**Figure 5:**
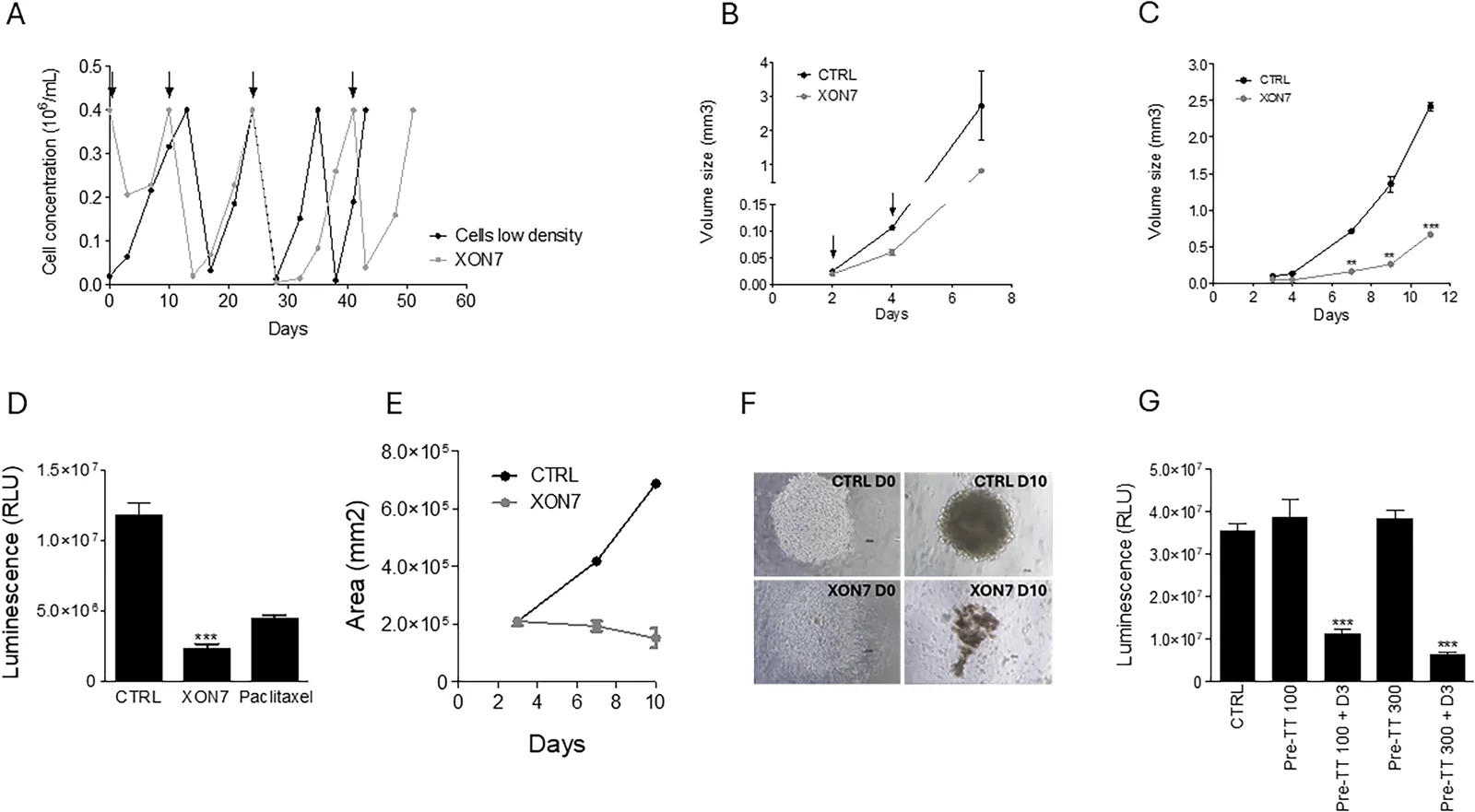
*In vitro* repeated XON7 treatment induces sustained cytotoxic effects without resistance emergence and remains active in 3D tumor models. **(A)** In KMS-12-BM multiple myeloma cells, repeated XON7 treatments (10 µg/mL, treatments indicated by the black arrows) induced progressive cytotoxicity with no evidence of resistance. **(B, C)** In 3D spheroid models, XON7 reduced tumor growth in HCT-116 colon cancer (43.5% and 69.6% volume reduction after treatments on days 2 and 4) and A549 NSCLC (≈70% reduction maintained until day 11 after a single treatment). **(D)** In MDA-MB-231 TNBC spheroids, XON7 (300 µg/mL) markedly inhibited growth, and repeated dosing (days 3 and 7) decreased viability beyond paclitaxel 6µM **(E, F)** Pretreatment of MDA-MB-231 cells (300 µg/mL, 24 h) impaired spheroid-initiating capacity, shown by the absence of compact structures. **(G)** In HCT-116 cells, pretreatment with XON7 (100–300 µg/mL) did not affect spheroid initiation but sensitized cells to retreatment, enhancing cytotoxicity (−70% and −85% viability). Data are expressed as mean ± SEM (n=3). Statistical analysis: One-way anova, *post-hoc* test Fischer, **p < 0.01, ***p < 0.001.

### XON7 suppresses tumor growth and metastasis *in vivo*

The anti-tumor activity of XON7 was next evaluated in xenograft models of NSCLC (A549), prostate cancer (LNCaP), colon cancer (HCT-116), and multiple myeloma (KMS-12-BM). Treatment was initiated when tumors reached approximately 50 mm³ and continued over 28 days. XON7 significantly inhibited tumor progression in all models. At day 28, reduction of tumor growth reached 57% in NSCLC (**Figure 6A**), 84.5% in prostate cancer (p<0.05) (**Figure 6B**), 60.2% in colon cancer (p<0.05) (**Figure 6C**), and 82.5% in the myeloma model (p<0.01) (**Figure 6D**), compared to untreated controls. In the colon model, tumor volume remained stable following treatment discontinuation, with median size decreasing further by D35 (81.5% reduction vs. controls), indicating a prolonged effect. In myeloma-bearing mice, tumor regrowth occurred post-treatment but remained moderate compared to untreated animals. To further assess anti-tumor activity in preclinically relevant settings, XON7 was tested in two dedifferentiated liposarcoma PDX models. Treatments were initiated when tumors reached 150–200 mm^3^. XON7 significantly delayed tumor growth in the CTG-2309 model (**Figure 6E**) and in the CTG-0910 model (**Figure 6F**) with sustained inhibition observed throughout the treatment period. The activity of XON7 was then assessed in two complementary orthotopic and metastatic rat xenograft models of TNBC using MDA-MB-231-Luc cells (**Figure 7**). In the orthotopic setting (injection in the mammary fat pad), XON7 treatment reduced primary tumor growth by >40% (**Figure 7A**) and metastatic dissemination, with an abrogation of detectable lesions in liver and lungs (p<0.01) (**Figure 7B**). In the metastatic setting (intravenous injection), XON7 significantly decreased metastatic burden as revealed by a near-complete suppression of metastatic signals in XON7-treated animals, after a whole-body bioluminescence imaging (**Figure 7C**). Quantification confirmed a significant reduction in lung metastases compared with controls (p<0.01; **Figure 7D**).

**Figure 6:**
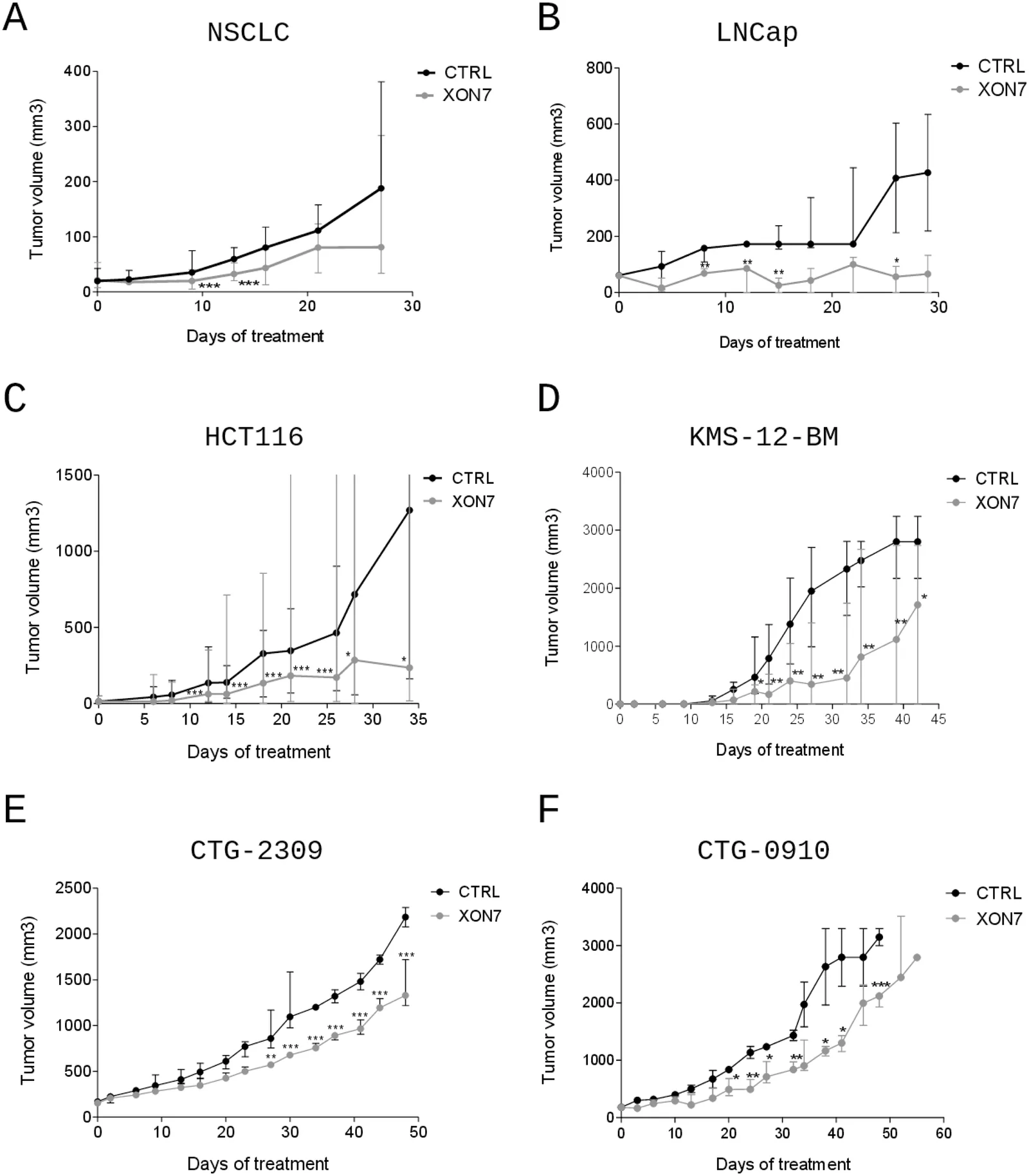
Anti-tumor efficacy of XON7 in murine xenograft and liposarcoma PDX models. Tumor growth in murine xenograft models of **(A)** NSCLC (A549 cell line), **(B)** prostate cancer (LnCap cell line), **(C)** colon cancer (HCT-116 cell line), and **(D)** myeloma (KMS-12-BM cell line) treated with XON7 (35 mg/kg, twice weekly) or untreated (CTRL). XON7 treatment began when tumors reached 50 mm³ and continued until D28. **(E, F)** In dedifferentiated liposarcoma PDX models, XON7 (40 mg/kg, intraperitoneal injection., twice weekly) significantly delayed tumor progression compared with controls. All data are shown as median ± SEM. Group sizes: n =10 per group. Statistical analysis: One-way anova, *post-hoc* test Fischer, *p < 0.05, **p < 0.01, ***p < 0.001.

**Figure 7:**
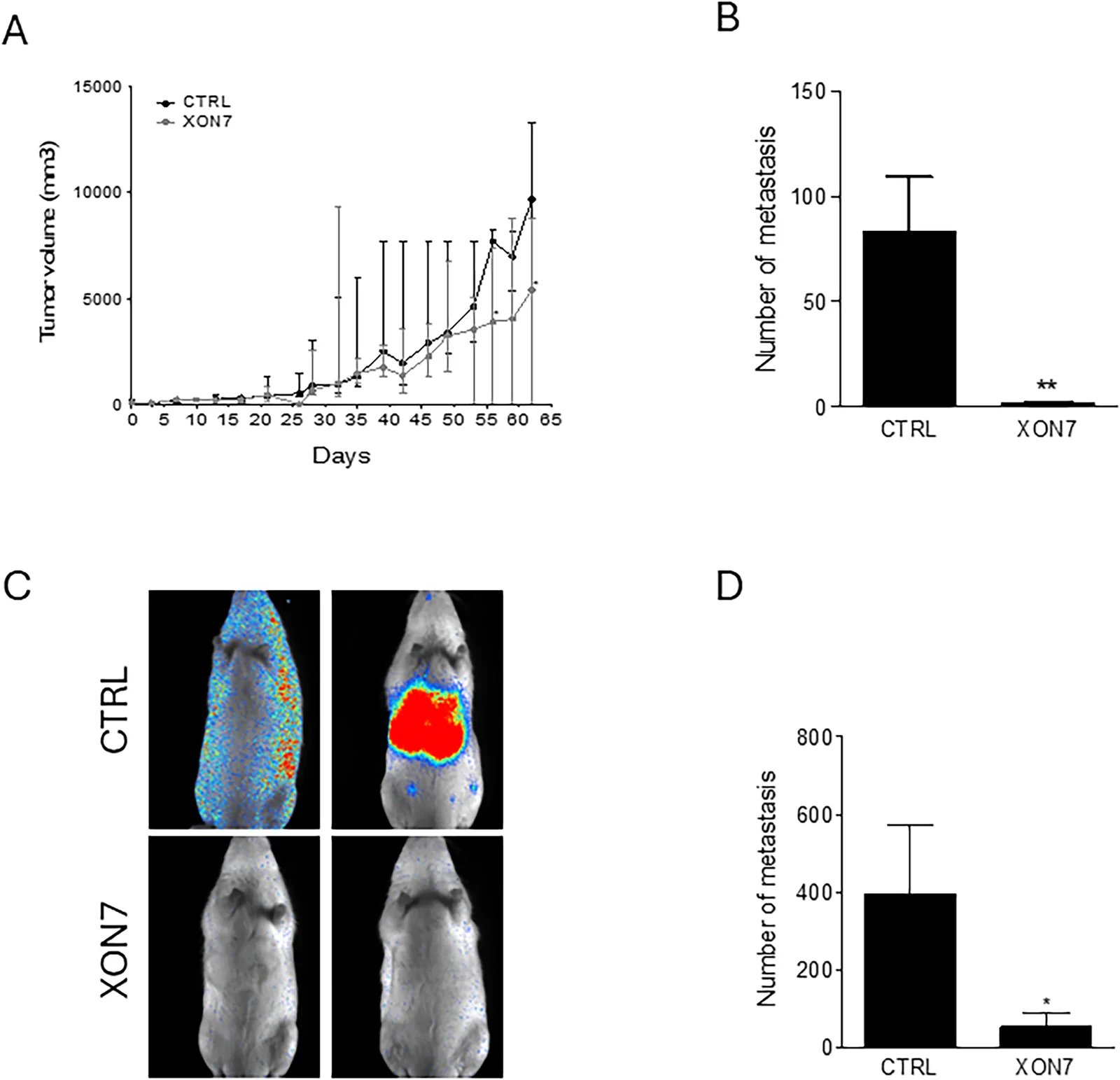
Anti-tumor and anti-metastatic activity of XON7 in TNBC rat metastatic models.**(A)** Orthotopic TNBC xenografts were established with bioluminescent MDA-MB-231-Luc cells and treated with XON7 (40 mg/kg, twice weekly, n=6) or untreated (CTRL, n=5). **(B)** Quantification of metastases in orthotopic models. **(C)** Bioluminescent whole-body imaging following intravenous injection of MDA-MB-231-Luc cells and luciferin detection, showing widespread metastatic burden in controls versus minimal signal in XON7-treated rats. **(D)** Quantification of lung metastases in the metastatic model (n=5). Data are shown as median ± SEM (n=7); One-way anova *post-hoc* Test Fischer *p < 0.05, **p < 0.01.

### Combination with PD-1 blockade enhances tumor control and modulates macrophage polarization

To explore XON7 immunomodulatory potential in combination with immune checkpoint inhibitors, we used a human lung cancer xenograft model in the chick chorioallantoic membrane (CAM). Treatment with suboptimal doses of XON7 or anti–PD-1 alone did not significantly reduce tumor burden. However, the combination of the two drugs led to a significant decrease in tumor weight (**Figure 8A**), demonstrating synergistic anti-tumor activity (Bliss synergy score 14%). Quantitative PCR analysis of human Alu sequences in CAM and brain samples revealed a significant reduction in metastatic dissemination with the XON7/anti-PD1 combination (Bliss score 72%), not observed with monotherapies (**Figure 8B**). RT-qPCR profiling of immune markers showed a significant increase in the M1/M2 macrophage ratio (p<0.05), suggesting macrophage polarization toward an anti-tumor phenotype under combination treatment (Bliss score 418%) (**Figure 8C**).

**Figure 8:**
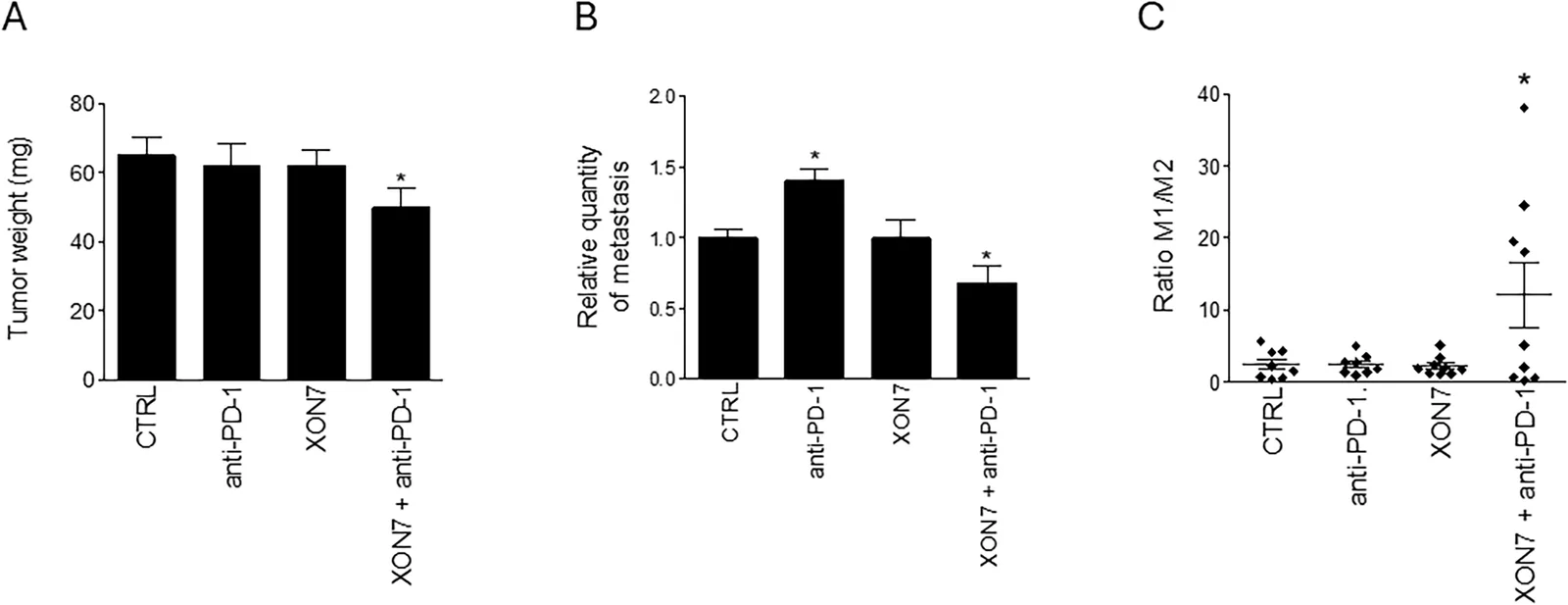
Anti-tumor activity in human tumor xenograft in chicken egg model (CAM). **(A)** Tumor growth, measured as tumor weight (mg) on day E18. CTRL n=17, anti-PD-1 n=14, XON7 n=16, XON7 + anti-PD-1 n=15 – Mean and SEM – One-way Anova – *Post hoc* test Fischer *p<0.05. **(B)** Metastatic burden, assessed as the relative quantity of metastases using qPCR analysis of Human Alu sequences in the CAM and brain samples. CTRL n=7, anti-PD-1 n=8, XON7 n=8, XON7 + anti-PD-1 n=7 – Mean and sem – One-way Anova – *Post hoc* test Fischer *p<0.05. **(C)** M1/M2 macrophage ratio determined using RT-qPCR analysis of M1 markers (iNOS2, CD86, CD80) and the M2 marker (CCL17). CTRL n=7, anti-PD-1 n=8, XON7 n=9, XON7 + anti-PD-1 n=8 – Mean and SEM – One-way Anova, *post hoc* test Fischer *p<0.05.

## Discussion

Monoclonal antibody therapies have substantially transformed oncology. Yet durable responses remain limited in many solid tumors due to antigen loss, pathway redundancy, and tumor heterogeneity. Increasing evidence indicates that tumors frequently escape single-target therapies through clonal selection or compensatory signaling networks (19–27). In this context, strategies capable of simultaneously targeting multiple tumor-associated pathways, such as bispecific antibodies, multispecific formats, and antibody combinations designed to address tumor complexity have attracted growing interest (1–3).

In this study, we investigated whether coordinated multi-antigen targeting through XON7, a defined GH-pAb preparation, could provide integrated anti-tumor activity. XON7 displayed broad cytotoxic activity across a diverse panel of tumor cells and models representing multiple cancer types. While several malignancies showed strong sensitivity, other models exhibited more heterogeneous responses. Such variability likely reflects differences in antigen expression, pathway dependency, and intrinsic tumor biology, which are well-recognized features of heterogeneous solid tumors. These findings further support the rationale for multi-antigen targeting strategies capable of addressing tumor diversity. Importantly, XON7 maintained biological activity in more complex experimental systems, including three-dimensional tumor spheroids and repeated treatment settings. In these models, sustained anti-tumor effects were observed without evidence of resistance emergence. The anti-tumor activity of XON7 was further confirmed *in vivo* across several xenograft models, including solid tumors, hematologic malignancies, and patient-derived xenograft systems. Through these models, treatment consistently reduced tumor growth and, in metastatic settings, markedly limited tumor dissemination. These results indicate that the biological effects observed *in vitro* translate into measurable anti-tumor activity *in vivo* across distinct tumor contexts.

Although XON7 recognized a broad variety of tumor cells, it showed no interaction with normal tissues or cells. Safety of XON7 was further confirmed in a GLP toxicology study (2 XON7 injections, 60mg/kg) conducted with common marmoset (*Callithrix jacchus*). No difference in body weight gain and food consumption was observed between treated and control groups. There were no cardiovascular (arrhythmia) or ophthalmological findings and no changes in hematology, coagulation, blood chemistry and urine parameters in XON7 group. Altogether, these results indicate that XON7 exhibits reproducible multi-target tumor binding with selective reactivity toward malignant tissues. Hence, safety, PK and anti-tumor efficacy of XON7 are currently evaluated in a first-in-human trial in patients with solid tumor (NCT06154291). Preliminary PK data collected from this trial confirmed the pertinence of the XON7 concentrations (100µg/ml and 300µg/ml) tested *in vitro* (14).

Proteome-scale screening identified three predominant tumor-associated targets of XON7: SLC3A2, CKAP4, and Annexin A2. Each protein has been independently implicated in tumor progression and cancer cell survival. SLC3A2 regulates amino acid transport and has been linked to tumor growth and metabolic adaptation in multiple cancer types (28–30). It has been validated as a high-value target with next-generation bispecific antibodies and antibody drug conjugates, demonstrating potent activity in preclinical models (31, 32), and is the therapeutic target of IGN523 mAb, tested in phase I trial in subjects with relapsed or refractory AML (NCT02040506 and (33)). CKAP4 functions as a receptor for Dickkopf-1 and activatesPI3K/AKT signaling pathway, promoting tumor cell proliferation and survival (34). Annexin A2 has been widely associated with tumor invasion, angiogenesis, and regulation of the tumor microenvironment, including macrophage recruitment and metastatic dissemination (35). The concurrent recognition of these proteins therefore suggests that XON7 interferes with complementary oncogenic pathways involved in tumor amino acid metabolism, survival signaling, and tumor–microenvironment interactions. Noteworthy, functional experiments revealed that the full anti-proliferative activity of XON7 depended on the concomitant presence of antibodies against SLC3A2, annexin A2 and CKAP4. The three proteins contribute to cell proliferation and tumor growth through the activation of various signaling pathways such as mTOR, PI3K/AKT, beta-catenin or ERK (34, 36–39). Development of compensation mechanisms within tumor cells probably developed when one target is left untouched. This highlights the potential of tumor cells to escape single target treatments and strengthen the rational of polyclonal antibody in cancer immunotherapy. As a key component of the xc^-^ cystine/glutamate antiporter system, SLC3A2 has been linked with immunogenic cell death through the release of DAMP such as HMGB1 (18, 40–42). We investigated the release of HMGB1 with XON7 or the anti-SLC3A2-positive or negative fractions. We observed liberation of HMGB1 by MDA-MB-231 cells treated with XON7 or the anti-SLC3A2 specific fraction. Anti-SLC3A2 fraction on its own did did not induce cell cytotoxicity, suggesting an active mechanism for HMGB1 release, as already described (43). Thus, beyond cytotoxicity, release of DAMP probably constitutes indirect mechanism of XON7 activity. The potential interaction between XON7-mediated tumor damage and the immune microenvironment was further explored in a CAM-based lung cancer model. In an experimental setting where XON7 and anti-PD1 under suboptimal dosing conditions produced limited effects, the combination of both treatments resulted in improved tumor control, reduction of invasion and metastasis, and macrophage polarization toward a M1-like phenotype. Thus, as shown with annexin A2 inhibition (44), cell stress and reprogramming of tumor immune microenvironment by XON7 may sensitize tumor cells to anti-PD1 therapy. Altogether, these results show that XON7 activity relies on the combined effects of distinct antibody populations and support a polyclonal mechanism that enhances anti-tumor efficacy by concurrently disrupting multiple tumor-associated pathways.

Our study has several limitations. Although depletion experiments support the importance of multi-antigen engagement, the respective contribution of individual specificities within the polyclonal preparation remains to be defined. Furthermore, our mechanistic investigation focused on 3 major targets of XON7. The contributions of other antibody populations within XON7 has not been investigated. Other markers of tumor cell stress and DAMP have been described (41, 42, 45), the extent to which these signals contribute to adaptive immune activation *in vivo* warrants further investigation. Finally, the CAM model provides a simplified immune environment (46), and future studies in fully immunocompetent systems will be required to better understand how multi-target polyclonal responses interact with host immunity.

Taken together, these findings support the concept that multi-antigen targeting through polyclonal antibody preparations may represent an alternative strategy to address tumor heterogeneity and signaling redundancy. While mAbs and bispecific antibodies represent obvious therapeutic modalities for their precision and reproducibility, our results argue for a reassessment of defined GH-pAb as viable therapeutic tools. Their capacity to integrate multiple functions (tumor targeting, immune engagement, pathway interference, induction of cellular and immunogenic stress), along with favorable safety profile (47–49) may prove particularly advantageous in heterogeneous and immunologically “cold” tumors. Our findings position XON7 as a promising multitarget immunotherapeutic with the potential to overcome key limitations of current monoclonal and bispecific strategies in oncology.

## Data Availability

The raw data supporting the conclusions of this article will be made available by the authors, without undue reservation.

## Supplementary material

Supplementary Figure 1 Analysis of SLC3A2, CKAP4 and AnnexinA2 depleted/specific fractions. Binding of each fraction against recombinant SLC3A2, AKAP4 and ANXA2 was investigated by ELISA. Recombinant SLC3A2, CKAP4 and AnnexinA2 (1 µg/mL, 100µl) were immobilized by overnight incubation at 4 °C on high-binding ELISA plates (Nest Scientific Inc., Woodbridge, NJ, USA). Wells were then washed with PBS containing 0.05% Tween 20 and saturated for 1 h at room temperature with PBS containing 0.05% Tween 20 and 2% bovine serum albumin (BSA) (Sigma-Aldrich, St Quentin Fallavier, France). XON7 fractions (100 µL in PBS/Tween/BSA) were added for 2 h at room temperature. Bound antibodies were detected with HRP-conjugated protein A (Sigma-Aldrich) and binding was revealed by TMB (Sigma-Aldrich). The reaction was stopped with 1 M H2SO4, and OD was measured at 450 nm (with a reference at 600 nm) with a microplate reader (Tecan, Männedorf, Switzerland).

Supplementary Figure 2 Expression of *SLC3A2*, *CKAP4* and *ANXA2* in various human cancer types. The TIMER database was used to investigate the expression of *SLC3A2*, *CKAP4* and *ANXA2* in 39 cancer types compared to normal tissues (*p<0.05; **p<0.01; ***p<0.005). ACC, adrenocortical carcinoma; BLCA, bladder urothelial carcinoma; BRCA, breast invasive carcinoma; CESC, cervical squamous cell carcinoma and endocervical adenocarcinoma; CHOL, cholangiocarcinoma; COAD, colon adenocarcinoma; DLBC, lymphoid neoplasm diffuse large B-cell lymphoma; ESCA, esophageal carcinoma; GBM, glioblastoma multiforme; HNSC, head and neck squamous cell carcinoma; HPV, human papilloma virus; KICH, kidney chromophobe; KIRC, kidney renal clear cell carcinoma; KIRP, kidney renal papillary cell carcinoma; LAML, acute myeloid leukemia; LGG, lower grade glioma; LIHC, liver hepatocellular carcinoma; LUAD, lung adenocarcinoma; LUSC, lung squamous cell carcinoma; MESO, Mesothelioma; OV, ovarian serous cystadenocarcinoma; PAAD, pancreatic adenocarcinoma; PCPG, pheochromocytoma and paraganglioma; PRAD, prostate adenocarcinoma; READ, rectum adenocarcinoma; SARC, sarcoma; SKCM, skin cutaneous melanoma; STAD, stomach adenocarcinoma; TGCT, testicular germ cell tumors; THCA, thyroid carcinoma; THYM, thymoma; UCEC, uterine corpus endometrial carcinoma; UCS, uterine carcinosarcoma; UVM, uveal melanoma.

Supplementary Figure 3 Clinical relevance of SLC3A2, ANXA2 and CKAP4 expression in cancer patients. **(A)** Heatmap generated using the TIMER platform, showing the association between SLC3A2, ANXA2 or CKAP4 expression and clinical outcome across cancer types. High Z-score is associated with poor prognosis. **(B)** Kaplan Meyer analyses generated using the GEPIA platform of the relationship between SLC3A2/CKAP4/ANXA2 gene set expression (quartile cutoff) and overall survival (OS) in BLCA, HSNC, LIHG, LUAD, LGG or mesothelioma patients.

Supplementary Figure 4 Multi-target recognition and batch-to-batch consistency of XON7. ELISA assays showed reproducible binding of three independent production batches of XON7 (Batch #1, Batch #2, Batch #3) to recombinant SLC3A2, CKAP4, and Annexin A2. Data represent mean ± SEM of triplicates.

Supplementary Figure 5 Reproductible *in vitro* anti-tumoral potency of XON7 batches across a panel of tumor cell lines. The CDC activity of three different batches of XON7 (Batch #1, Batch #2 and Batch #3) was evaluated after 24h of incubation with rabbit complement and increasing antibody concentrations. The tumor cell lines tested were SK-MEL-30 (melanoma), Hep-G2 (hepatocellular carcinoma), HCT-116 (colorectal cancer), MDA-MB-231 (Triple negative breast cancer), LNCaP (prostate cancer), and KMS-12-BM (multiple myeloma). Results demonstrate consistent cytotoxic efficacy across all three batches, confirming batch reproducibility. Data are ex-pressed as the percentage of cytotoxicity (n=3, mean ± SEM.
